# Engineering of *Escherichia coli* Glyceraldehyde-3-Phosphate Dehydrogenase with Dual NAD^+^/NADP^+^ Cofactor Specificity for Improving Amino Acid Production

**DOI:** 10.3390/microorganisms10050976

**Published:** 2022-05-06

**Authors:** Ekaterina A. Slivinskaya, Natalia S. Plekhanova, Irina B. Altman, Tatiana A. Yampolskaya

**Affiliations:** Ajinomoto-Genetika Research Institute, 117545 Moscow, Russia; plekhanovans@mail.ru (N.S.P.); irina_altman@agri.ru (I.B.A.); tatiana_yampolskaya@agri.ru (T.A.Y.)

**Keywords:** glyceraldehyde-3-phosphate dehydrogenase, NAD^+^, NADP^+^, cofactor specificity, l-threonine, l-lysine, l-proline

## Abstract

Glyceraldehyde-3-phosphate dehydrogenase (GAPDH) is a key enzyme in the central metabolism of microbial cells. GAPDHs differ in cofactor specificity and use NAD^+^, NADP^+^, or both cofactors, reducing them to NADH and NADPH, respectively. Sufficient NADPH supply is one of the critical factors required for synthesis of the amino acids l-lysine, l-threonine, and l-proline in industrially important *Escherichia coli*-based producer strains. *E. coli* cells have NAD^+^-dependent glycolytic GAPDH. One reasonable approach to increase NADPH formation in cells is to change the specificity of the GAPDH from NAD^+^ to NADP^+^. In this study, we modified the cofactor specificity of *E. coli* GAPDH by amino acid substitutions at positions 34, 188 and 189. Several mutant enzymes with dual NAD^+^/NADP^+^ cofactor specificity were obtained, and their kinetic parameters were determined. Overexpression of the genes encoding the resulting mutant GAPDHs with dual cofactor specificity in cells of l-lysine-, l-threonine-, and l-proline-producing *E. coli* strains led to a marked increase in the accumulation of the corresponding amino acid in the culture medium. This effect was more pronounced when cultivating on xylose as a carbon source. Other possible applications of the mutant enzymes are discussed.

## 1. Introduction

**Figure 1:**
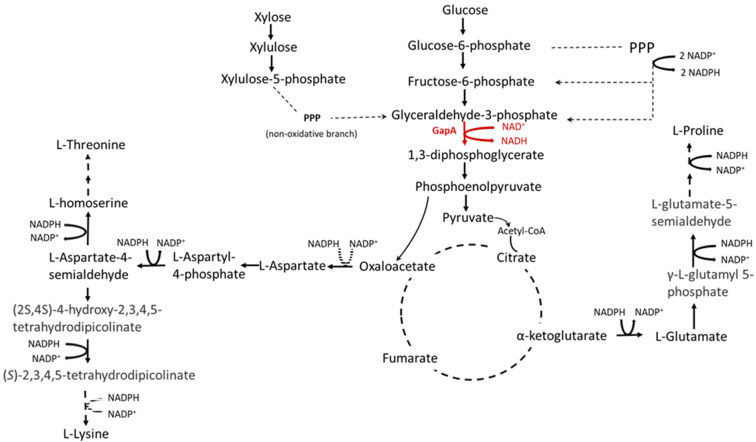
Schematic representation of some amino acids of l-aspartate and l-glutamate family biosynthesis in *E. coli*.

The most significant redox cofactors in the cell metabolism, NADH/NAD^+^ and NADPH/NADP^+^, provide both abatement power for energy-conserving redox reactions and also participate as electron acceptors in metabolic reactions [1,2]. However, NADH/NAD^+^ is preferentially used for catalyzing substrate oxidation, whereas NADPH/NADP^+^ is mainly used for catalyzing substrate reduction. Hereby, balancing the oxidation–reduction status of NADH/NAD^+^ and NADPH/NADP^+^ is decisive for both anabolism and catabolism and is very important for cell growth and synthesis of several industrially important products, such as some amino acids [3]. Amino acids in the l-aspartate family, such as l-lysine and l-threonine, are essential amino acids required for animal and human nutrition. They must be present in food and feed, which, in many cases, is realized by supplementation with pure substances [4]. l-proline is a non-essential amino acid; however, currently, it is widely used in health foods. Additionally, l-proline is a good osmoprotectant. These amino acids are mainly produced by various prokaryotic producers, which were constructed by conventional mutagenesis-screening techniques or by rational design through genetic engineering [5,6]. Approximately 800,000 tons of l-lysine, 230,000 tons of l-threonine, and 100 tons of l-proline are currently produced microbiologically worldwide each year (feedlot.ru, vimeo.com 30 March 2022). *Escherichia coli* strains are one of the most important industrial producers of amino acids. For efficient production of l-lysine, l-threonine, and l-proline, the supply of the NADPH cofactor is a critical factor. For the synthesis of 1 mol of l-lysine and 1 mol of l-threonine from oxaloacetate, an *E. coli* cell requires 4 and 3 mol of NADPH, respectively. For 1 mol of l-proline biosynthesis from α-ketoglutarate, 3 mol of NADPH should be supplied (Figure 1). Thus, many studies have been focused on engineering the metabolism of NADPH to improve the respective amino acid-producing strains [7,8].

Studies on the kinetics of pyridine nucleotide metabolism in *E. coli* have shown that the ratio of NADP^+^ to NAD^+^ mainly depends on the rate of their mutual transformation provided by membrane-bound proton-translocating transhydrogenase PntAB [9]. In addition, the main sources of NADPH in the cell are NADP^+^-dependent dehydrogenases of the oxidative branch of the pentose phosphate pathway (PPP), which synthesize 2 mol of NADPH while utilizing 1 mol of glucose, and isocitrate dehydrogenase, which is involved in the tricarboxylic acid cycle. According to the literature, several methods to increase the supply of this cofactor are known: redirection of the main carbon flux of glucose-6-phosphate from the Embden–Meyerhoff–Parnas pathway (EMP) to PPP due to inactivation of phosphoglucoisomerase [10], overexpression of genes encoding NADP^+^-dependent glucose-6-phosphate-1-dehydrogenase and 6-phosphogluconate-2-dehydrogenase [11], and expression in the cell of heterologous genes encoding NADP^+^-dependent proteins [7,12].

One promising method is to replace the cofactor specificity of some NAD^+^-dependent enzymes with a NADP^+^-dependent one [13]. One of the candidates for such modification is glyceraldehyde-3-phosphate dehydrogenase (GAPDH), an enzyme of the central pathway of carbon metabolism that catalyzes the oxidation of D-glyceraldehyde-3-phosphate (G-3P) to 1,3-bisphosphoglycerate with the reduction of NAD^+^ to NADH. Three types of GAPDH with different subcellular localizations and functions in the cell have been identified. Non-phosphorylating NADP^+^-dependent glyceraldehyde 3-phosphate dehydrogenase (GapN; EC 1.2.1.9), which catalyzes the irreversible oxidation of G-3P to 3-phosphoglycerate with the reduction of NADP^+^ to NADPH, has been found in photosynthetic eukaryotes and in some Gram-positive bacteria, such as *Streptococcus* [14] and *Clostridium* [15] species. This enzyme is characterized by a low specific activity. Enzymes of the following two classes belong to phosphorylating GAPDH and use inorganic phosphate as a substrate in the reversible oxidation reaction of G-3P to 1,3-diphosphoglycerate. GAPDHs of the second type, which are widely distributed in nature as so-called photosynthetic GAPDHs, are found in organisms with oxidative photosynthesis, such as cyanobacteria, green and red algae, and higher plants [16]. They are localized in the stroma of chloroplasts and are involved in photosynthetic assimilation of CO_2_ in the reducing branch of the PPP. This class includes enzymes mainly having dual NAD^+^/NADP^+^ cofactor specificity. The proteins of the third type of GAPDH are localized in the cytoplasm and play a major role in glycolysis and gluconeogenesis. In most microorganisms and in *E. coli*, this enzyme uses NAD^+^ as a cofactor (EC 1.2.1.12). However, a third type of GAPDH with double cofactor specificity has now been found, for example, *Kluyveromyces lactis* GAPDH (EC 1.2.1.59) [17]. Some organisms, such as *Bacillus subtilis* and *Bacillus stearothermophilus*, have two GAPDH isoenzymes, GapA and GapB, involved in glycolysis and gluconeogenesis, respectively, and they use NAD^+^ (EC 1.2.1.12) and NADP^+^ (EC 1.2.1.13) as cofactors, respectively [18]. Amino acid sequences of glycolytic GAPDHs are highly conserved. Based on the analysis of enzymes with different cofactor specificities, several authors have identified the main amino acid sequences responsible for the interaction of the protein molecule with NAD^+^/NADP^+^: the region of the 30th amino acid residue and the region between the 180th and 190th amino acid residues [19]. Previously, attempts were made to change the cofactor specificity of the NAD^+^-dependent enzymes for *B. subtillis* and *B. stearothermophilus* by purposefully replacing the amino acid residues in the cofactor-binding center of the protein. Thus, it was shown that single substitutions of the aspartate residue at position 32 (D32E, D32A, D32N, D32G), leucine and proline residues at positions 187–188 (L187A, L187N/P188S), and their combinations in the sequence of the *B. subtilis* GAPDH protein led to the ability of the enzyme to use both cofactors. Nevertheless, the obtained proteins were characterized by reduced enzymatic activity [20,21]. The similar results were obtained in experiments on changing of the substrate specificity of *C. glutamicum* GAPDH [22]. The substitution of the aspartate residue at 35th position (D35G) as well as L36T, T37K and P192S allowed GAPDH to accept both NAD^+^ and NADP^+^. It is worth emphasizing that in all the above works, the presence of the aspartate residue in the catalytic center of the enzyme strictly prohibited the binding of NADP^+^.

Based on these facts we have defined the purpose of our work as the site-directed modification of GAPDH *E. coli* to modify the cofactor specificity and construct a mutant enzyme with NADP^+^-dependent activity to improve NADPH generation in cells. We demonstrate experimental evidence that the replacement of native NAD^+^-dependent GAPDH with NAD^+^/NADP^+^-dependent GAPDH improves cell growth, and production of target amino acids, l-lysine, l-threonine, and l-proline (as we assume due to an increase in the pool of NADPH in the cell).

## 2. Materials and Methods

### 2.1. Bacterial Strain, Plasmids, and Growth Conditions

The *E. coli* strain K-12 MG1655 and its derivatives were used in this study (Table 1). The plasmids used in the work are shown in Table 2. Rich Luria–Bertani (LB) medium and minimal medium M9 [23] supplemented with different carbon sources (0.2% glucose, or 1% ethanol, or 0.2% glycerol with the addition of other carbon sources) were used for cell cultivation. The cultures were grown at 37 °C with aeration, and growth was measured by the absorbance at 600 nm using a Shimadzu UV-1800 spectrophotometer. The antibiotics ampicillin (Amp, 100 µg/mL), kanamycin (Kan, 50 µg/mL), and chloramphenicol (Cm, 40 µg/mL) as well as the vitamin thiamine (5 µg/mL) and amino acids (50 µg/mL) were added, if needed.

### 2.2. Recombinant DNA Techniques

The *E. coli* strain transformation, obtaining of the P1vir phage, and transduction were carried out according to standard methods [27]. DNA purification was carried out using a Qiagen Miniprep kit and Qiagen DNA purification kit (Qiagen, Germantown, MD, USA). PCR was performed using a DNA thermal cycler (GeneAmp PCR System 9700, Applied Biosystems, Bedford, MA, USA) using AccuTag™ DNA polymerase (USA) [28]. The restriction endonucleases were purchased from Fermentas (Thermo Fisher Scientific, Waltham, MA, USA).

### 2.3. Strains Construction

The primer sequences used in this study are listed in Table S1. All primers were designed based on the genomic sequences of *E. coli* and purchased from Syntol, Moscow, Russia.

The strain MG1655 Cm-ymfR::gapA with the second copy *gapA* gene instead of the *ymfR* gene was constructed using the lambda Red integration procedure [25] by substitution of the *ymfR* gene with the *cat-gapA* cassette. Gene *gapA* inactivation in the *E. coli* chromosome was carried out by the lambda Red integration procedure by substitution of the *gapA* gene in the native locus of the chromosome by the *kan* gene (providing kanamycine resistance) using the plasmids pKD46 [25] and pMW119-attL-Km-attR [26]. The cassette Δ*gapA*::*kan* was constructed using oligonucleotides P1 and P2 (Table S1). Deletion of *gapA* was verified by PCR using oligonucleotides P3 and P4. The strain YA1461 Δ*gapA*::*kan* was obtained by transduction of the Δ*gapA*::*kan* cassette into strain YA1461 carrying the mutant alcohol dehydrogenase. Selection of transductants was carried out on plates with ethanol as a carbon source and kanamycin.

The cassette containing an additional copy of the *gapA* gene was constructed by overlapping the PCR of two fragments: (1) the fragment carrying the chloramphenicol-resistant gene from the pMW119-attLλ-cat-attRλ plasmid, obtained using oligonucleotides P5 and P7, and (2) the *gapA* gene with a native regulatory region, obtained using MG1655 chromosomal DNA as a template and oligonucleotides P6 and P8. The obtained fragments and oligonucleotides P5 and P8 were used for construction of the full-size *Cm-ymfR::gapA* cassette. Introduction of the additional copy of the *gapA* gene into the chromosome was carried out by lambda Red integration; the constructed strains were verified by PCR using oligonucleotides P9 and P10.

**Figure 2:**
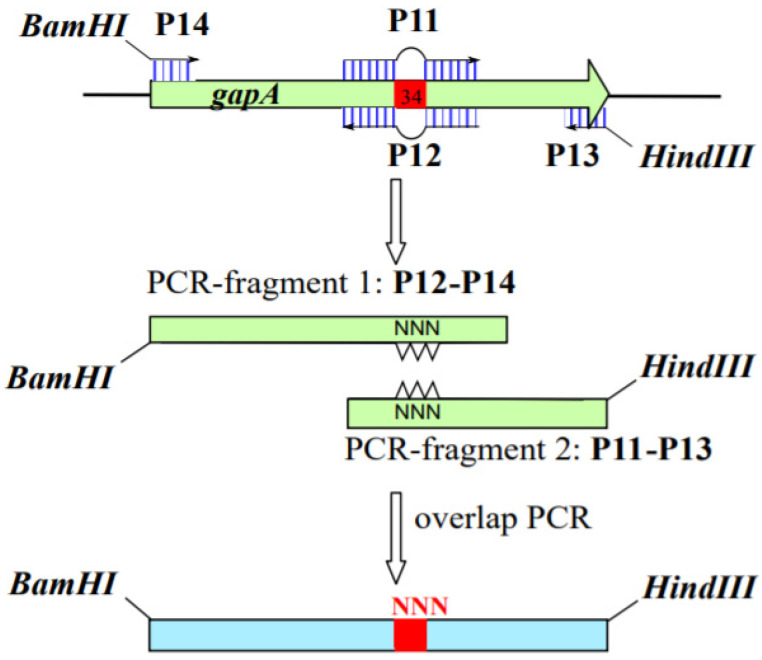
Scheme of construction of the mutant *gapA* gene with random mutation at position 34.

The wild type *gapA* gene was obtained by PCR using primers P3 and P4 and genomic DNA of *E. coli* MG1655 as a template according to the standard procedure [23] and then cloned on the plasmid pKK233-2 yielding the plasmid pKK-gapA^wt^. The pKK-gapA^D34nnn^ plasmids carrying the *gapA* gene encoding GAPDH with random mutation of the 34th amino acid were constructed by three-step PCR using primers P11–P14 (Table S1, Figure 2) and genomic DNA of MG1655 as a template.

First, a DNA fragment was obtained using primers P12 and P14. Primer P12 was homologous to the 130–102-bp and 99–71-bp regions of the *gapA* gene, between which the random sequence *nnn* corresponding to 102–100 nucleotides of the gene was introduced. Primer P14 was homologous to the 5′-end of the *P_gapA_* promoter and contains the restriction site *Bam*HI for further cloning into the plasmid. At the next step, the second DNA fragment was obtained using primers P11 and P13. Primer P11 was homologous to the 71–99-bp and 103–130-bp regions of the *gapA* gene, between which the random sequence *nnn* corresponding to 102–100 nucleotides of the gene was introduced. Primer P13 was homologous to the 3′-end of the *gapA* gene and contains the restriction site *Hind*III for further cloning. The obtained DNA fragments were used as a template for the synthesis of the full-size *gapA* gene using overlapping PCR with primers P13 and P14. The target fragment was treated with *Bam*HI and *Hind*III and cloned into the similarly treated vector pKK233-2. Thus, the library of pKK-gapA^34mut^ plasmids containing the mutant *gapA* gene was obtained. Plasmids were transformed into the *E. coli* YA1461 ΔgapA::kan strain with selection of transformants on M9 medium with 0.4% glucose, 2% ethanol, and 0.1% yeast extract supplemented with ampicillin. Selected colonies were tested for growth on complete medium with ampicillin.

The plasmids carrying mutant *gapA* genes with substituted residues G188 and P189 were constructed according to the procedure described previously [29] using primers P15–P18. For introduction of these mutations into the amino acid sequence of GAPDH, the corresponding primer pairs were used.

Each pair of primers contained non-overlapping sequences on the 3′-end and the primer–primer complementary (overlapping) sequences at the 5′ end. Primers P15 and P16 were used to replace G188 by T (*ggc* by *acc*) and P189 by K (*ccg* by *aaa*), primers P17 and P18 were used to replace G188 by V (*ggc* by *acg*) and P189 by R (*ccg* by *cgt*). Mutant plasmids were tested using locus-specific PCR using the P13 and P19 primers to determine the original amino acid residues G188 and P189; P13 and P20 primers were used to determine the mutant amino acid residues T188 and K189; P13 and P21 were used to determine the mutant amino acid residues V188 and R189.

### 2.4. Enzyme Purification and Assay

*E. coli* strains were grown in liquid M9 or LB medium for 4–8 h at 37 °C to late log phase (up to an optical density, OD_600_ ≈ 2.0). Cells were collected by centrifugation, washed twice with 0.9% NaCl, suspended in buffer (100 mM Tris-HCl, 20 mM KCl, 0.5 mM EDTA, 2 mM DTT, pH 7.5), and sonicated on ice using an ultrasonic disruptor (MSE, Nuaillé, France). Cell debris was removed by centrifugation, and the resulting supernatant was provided for the enzyme assay. The protein concentration in the cell extracts was estimated via the Bradford procedure using a Bio-Rad Protein Assay (Bio-Rad Laboratories GmbH, Kabelsketal, Germany). All reagents used in the current work were purchased from Sigma, Ronkonkoma, NY, USA). Total protein electrophoresis in 15% polyacrylamide gel was performed in accordance with the procedure presented by Laemmli [30].

GAPDH activity was spectrophotometrically measured in cell extracts according to the procedure described previously [31] with addition of NAD^+^ or NADP^+^ (2 mM) as a cofactor. Purification of GAPDH for determination of the kinetic parameters was carried out by fractional precipitation with (NH_4_)_2_SO_4_. To purify the target protein, crude cell extract was treated with (NH_4_)_2_SO_4_ to yield 3 fractions: 0–60% (I), 60–75% (II) and 75–85% (III) of ammonium sulfate saturation. As shown in Figure S1, in fraction I the target protein band was almost absent, in fraction II it was strongly enhanced, and in fraction III an almost homogeneous GAPDH protein was obtained. Protein fraction III was used to evaluate the enzyme’s kinetic characteristics with respect to NAD^+^/NADP^+^. Calculation of the enzyme kinetic properties was performed using SigmaPlot version 10.0 (Systat Software, Inc., San Jose, CA, USA).

### 2.5. Evaluation of l-Amino Acid Accumulation in Culture Broth Using Test Tube Fermentation

Initially, the l-amino acid-producing strains were grown for 18–24 h at 37 °C on plates with l-agar containing ampicillin. To obtain the seed culture, the strains were grown in 20 × 200-mm test tubes containing 2 mL of LB on a rotary shaker (250 rpm) at 37 °C for 18 h. Next, 0.2 mL of seed (10%) was introduced into the fermentation medium. Fermentation was carried out in test tubes with 2 mL of fermentation medium. After cultivation, the amount of amino acid in the medium was determined by thin layer chromatography (Sorbfil plates). For amino acid detection, we used 1% ninhydrin solution in acetone (15%). Calculation of the amount of amino acid was performed on a scanning densitometer Camag^®^ Linomat 5 (Camag^®^, Muttenz, Schwitzerland). The optical density was measured using a Shimadzu UV1800 spectrophotometer (SHIMADZU, Kyoto, Japan).

l-lysine. Fermentation of l-lysine-producing strains was carried out at 37 °C for 48 h using a reciprocal shaker at an agitation speed of 240 rpm. The composition of the fermentation medium (g/L) was as follows: sugar (glucose or xylose)—40.0; (NH_4_)_2_SO_4_—24.0; K_2_HPO_4_—1.0; MgSO_4_·7H_2_O—1.0; FeSO_4_·7H_2_O—0.01; MnSO_4_·5H_2_O—0.01; Thiamine-HCl—0.0002; Yeast extract—2.0; CaCO_3_—30.0; pH = 7.0.

l-threonine. Fermentation of l-threonine-producing strains was carried out for 72 h at 32 °C with shaking at 240 rpm. The composition of the fermentation medium (g/L) was as follows: sugar (glucose or xylose)—80.0; (NH_4_)_2_SO_4_—22.0; NaCl—0.8; KH_2_PO_4_—2.0; MgSO_4_·7H_2_O—0.8; FeSO_4_·7H_2_O—0.02; MnSO_4_·5H_2_O—0.02; Thiamine–HCl—0.0002; Yeast extract—1.0; CaCO_3_—30.0; pH = 7.0

l-proline. Fermentation of l-proline-producing strains was carried out for 72 h at 32 °C with shaking at 240 rpm. The composition of the fermentation medium (g/L) was as follows: sugar (glucose or xylose)—60, (NH_4_)_2_SO_4_—25, KH_2_PO_4_—2, MgSO_4_·7H_2_O—1.0, l-Isoleucine—0.05; Thiamine–HCl—0.0001, CaCO_3_—25, pH = 7.2.

## 3. Results 

### 3.1. Construction of a Recipient Strain to Obtain the Mutant gapA Gene Library

The strain with the deleted *gapA* gene was necessary for selection of mutant *gapA*. Attempts to directly knock out the *gapA* gene in *E. coli* MG1655 were unsuccessful, which is consistent with earlier published data [32,33] that the deletion of the *gapA* gene makes *E. coli* cells practically incapable of growing on a rich medium, as well as on the minimal medium with glucose as the sole carbon source. It has been suggested that the strains with *gapA* deletion could grow on other carbon sources that are utilized via bypassing glycolysis, such as ethanol. Indeed, Δ*gapA*::*kan* mutants were successfully selected based on the strain YA1461, carrying a mutant *adhE* encoding an aerobically active ethanol dehydrogenase, on plates with ethanol as the carbon source; as a result, strain YA1461ΔgapA::kan was obtained. The construction of the desired strain was carried out in two stages. At the first stage, a second copy of the *gapA* gene with its own regulatory region was introduced into the *ymfR* locus of the MG1655 chromosome. *YmfR* is uncharacterized protein (putative phage terminase protein A family, e14 prophage). A number of studies and our experimental data have shown that the deletion of gene *ymfR* does not affect the phenotype and survival of *E. coli* cells. A two-fold increase in the enzymatic activity of GAPDH in recombinant clones with the cassette *ymfR::gapA-cat* confirmed the presence of a functional protein encoded by this gene (Table 3).

For inactivation of the native copy of *gapA*, *ΔgapA*::*kan* cassette was integrated into the resulting strain MG1655Cm-gapA::ymfR, and kanamycin-resistant clones were selected. The activity of GAPDH in cell extracts confirmed the deletion of the native copy of the gene in the chromosome (Table 3). Finally, the locus with cassette *ΔgapA::kan* was introduced into the chromosome of the YA1461 strain by P1 transduction. YA1461ΔgapA::kan clone breeding was carried out on a minimal medium with ethanol, glucose, and kanamycin. However, strain YA1461 ΔgapA::kan was also characterized by the weak growth on minimal media with ethanol. After testing the different compounds (Table S2), optimal conditions for strain cultivation were chosen: M9 medium with 0.4% glucose, 2% ethanol, and 0.1% yeast extract. The absence of GAPDH activity in cell extracts confirmed the inactivation of *gapA* on the chromosome of YA1461ΔgapA::kan (Table 3).

### 3.2. Construction of Mutant gapA Genes with Replacement in the 34th Position

As mentioned earlier, the amino acid sequences of glycolytic GAPDH prokaryotes and eukaryotes are characterized by a high degree of conservativeness. Thus, it was shown that the presence of an aspartate residue at position 32 in different species of *Bacillus* or in position 35 of *C. glutamicum* is directly related to the NAD^+^ specificity of the enzyme and excludes the binding of NADP^+^ [18,20,22]. At the same time, the presence of alanine or glycine at this position leads to an affinity for NADP^+^. According to the authors of these studies, changes in the amino acid sequence proteins, which are aimed at overcoming steric difficulties in binding the substrate or affecting the nature of the electrostatic interaction between amino acid residues in the conferment-binding centers, can lead to a change in the cofactor specificity of the enzyme. First, we tried to obtain NADP^+^-dependent *E. coli* GAPDH encoded by the gene *gapA* by replacing the aspartate residue at the 34th position of the protein chain (this position corresponds to the 32nd amino acid residue of *B. subtillis* and 35th residue in *C. glutamicum*) (Figure S2). The *gapA* gene was cloned into the pKK233-2 plasmid, and then, a set of mutant *gapA* genes with substitution in the codon encoding the 34th amino acid was obtained using random mutagenesis. Strain YA1461ΔgapA::kan was transformed using the obtained plasmids. Plasmids from strains capable of growing on LA plates (complementation of *gapA* deletion) were isolated and analyzed. A high pKK233-2 copy number provides a large amount of GapA protein in cells; on SDS-PAGE electrophoresis, we observed only one major band; this band correlated with the expected MW of *E. coli* GAPDH (approximately 34 kD) (Figure S3).

We found only five mutants that complemented GapA deficiency; the GAPDH activity assay revealed that four clones demonstrated double cofactor specificity. Sequence analysis of the corresponding plasmids showed that the change in cofactor specificity is a result of substitution of the aspartic acid residue by proline (*gac* to *ccc*), lysine (*gac* to *aaa*), alanine (*gac* to *gct*), or leucine (*gac* to *ctc*) in the 34th position of the amino acid sequence of GAPDH, respectively; replacement of aspartate on asparagine led to a decrease in NAD^+^-dependent activity without an increase in NADP^+^-dependent activity. A substitution of the aspartic acid residue with glycine was also obtained, but the plasmid with the mutant gene did not complement *gapA* deletion. We suggest that, unlike the mutant GapA^D35G^ from *C. glutamicum*, the mutant GapA^D32G^ of *E. coli* provides a low level of GAPDH activity.

The replacement of the aspartate residue with alanine, which provides the highest level of activity, was previously described by several authors as necessary for the binding of NADP^+^; however, the effects of the other three mutations on the binding of the cofactor are not known. In all four cases, the affinity for NADP^+^ was accompanied by a significant decrease in the total activity of GAPDH compared with the activity of the wild-type enzyme (Table 4). As shown in Figure S3, the amount of native and mutant proteins in the cell is approximately the same for all variants; this means that the replacement of aspartate in the 34th position leads to a decrease in the rate of the enzymatic reaction, and the low activity of GAPDH is characteristic of the enzyme itself.

### 3.3. Study of the Kinetic Characteristics of Mutant GAPDH 

For all mutant GAPDHs with double cofactor specificity, Michaelis–Menten kinetic values were estimated for both NAD^+^ and NADP^+^; they are represented in Table 5. As shown, all mutant enzymes with sole substitution in the 34th position retained the NAD^+^-dependent activity; however, affinity to NAD^+^ became drastically low. At the same time, acquired NADP^+^-dependent activity was characterized by a low specific activity and rather low affinity for NADP^+^.

**Figure 3:**
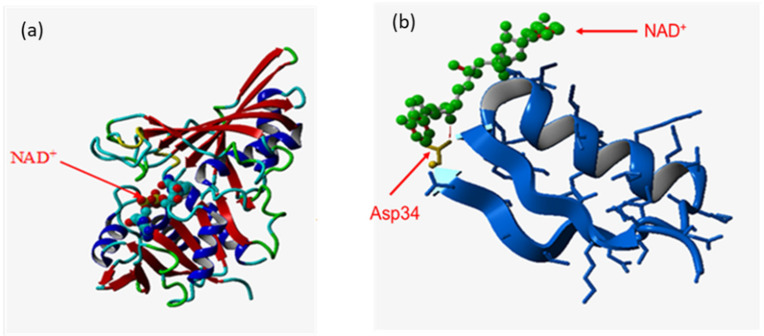
Ribbon diagram representation of the 3D structure of GAPDH *E. coli.* (**a**) a subunit of wild-type GAPDH with the specified position of the coenzyme-binding site. (**b**) wild type: fragment of 1 to 40 amino acid residues with a coenzyme-binding site. In yellow—residue D34, in green—NAD^+^.

Position 34 is located in the active site of the holoenzyme (Figure 3a) [34] at the edge of the pocket where the cofactor (NAD^+^ in the wild-type enzyme) attaches during the reaction. The aspartate residue in this position is crucial for NAD^+^-dependent GAPDH activity (Figure 3b): random mutagenesis of this position leads to loss (mutants unable to complement *gapA* deletion) or a drastic decrease in this kind of activity.

**Figure 4:**
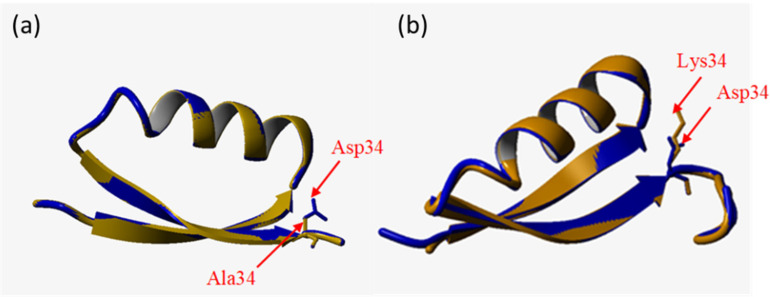
Comparison of the structure of coenzyme-binding centers of mutant GAPDH and the wild-type protein. (**a**) replacement of D34A: fragment of 1 to 40 amino acid residues. In blue—wild type, in yellow—mutant protein. (**b**) replacement of D34K: fragment of 1 to 40 amino acid residues. In blue—wild type, in yellow—mutant protein.

Moreover, replacement of D34 onto A (Figure 4a) or K (Figure 4b) provides NADP-dependent activity. According to the analysis of the 3D structure of GAPDH, replacement of acidic aspartate to non-polar alanine contributes to stabilization of hydrogen bonds with the phosphate group of NADPH and simultaneously increases the space for the coenzyme-binding pocket. As a result, mutant GAPDH shows a weak affinity to both cofactors. The positively charged lysine in position 34 interacts with the negatively charged NADP^+^ phosphate group. In this case, the cofactor can be held in the coenzyme-binding pocket, and the affinity of this D34K to NADP^+^ is sufficiently higher in comparison with that of D34A. Mutants D34A and D34K seemed to be the most promising in terms of kinetic parameters; however, further modification of GAPDH is required to improve the NADPH-dependent activity.

### 3.4. Construction of GAPDH Mutants with Several Substitutions

As shown above, replacement of aspartate in the 34th position of the GapA protein in *E. coli* allowed us to obtain enzymes with double NAD^+^/NADP^+^ cofactor specificity; however, mutant GAPDHs showed a drastic decrease in the NAD^+^-dependent activity and a low NADP^+^-dependent activity. To improve the NADP^+^-dependent activity, we constructed other modifications of the GapA protein. According to literature data [10,12,26], there are two additional amino acids that are important for NADP^+^ specificity in GAPDH, along with the 34th position. In *E. coli*, they are 188G and 189P (Figure S2). Based on an analysis of GapB of *B. subtilis* and *B. stearothermophilus,* S. Fillinger et al. demonstrated that the combination of two modifications of the 32th and 187th amino acid residues of GapA (corresponding to the *E. coli* 34th and 188th positions, respectively) improved the NADP^+^-depended enzyme activity in comparison with single substitution at the 32th position. Alignment of GapA in *E. coli* with the amino acid sequences of the NADP^+^-dependent proteins of *H. pylori, N. gonorrhoeae*, and *N. meningitides* revealed that the *E. coli* enzyme has 188G and 189P amino acid residues whereas proteins with NADP^+^ cofactor specificity have N, T, or V at the 187th position and K or R at the 189th position. Thus, we decided to replace 188G with 188N (based on homology with NADP^+^-dependent GapB in *B. subtilis),* 188G189P with 188T189K (based on homology with Gap2 in *H. pylori),* and 188G189P with 188V189R (based on homology with Gap2 in *N. meningitides)* in *E. coli* GapA protein sequence.

**Figure 5:**
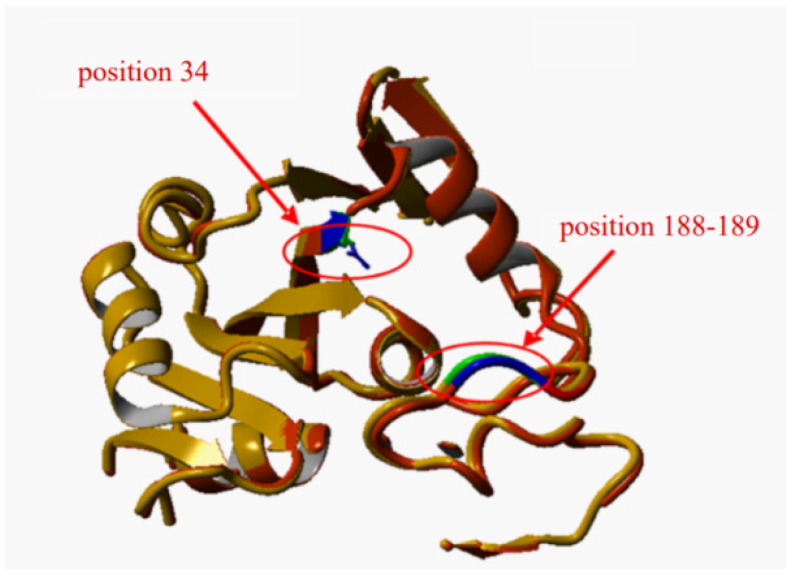
The mutual orientation of sites 34 and 188–189 in the N-terminal domain (fragment of 1 to 200 amino acid residues). In red—wild type D34; in yellow—mutant protein G188TP189K.

Site-directed mutagenesis was used for construction of the desired mutant proteins. As shown in Table 4, all mutant proteins with replacements at the 188 and 189 positions retained the NAD^+^-dependent activity as the wild type level or higher. Two mutants, GapA^G188TP189K^ and GapA^G188VP189R^, which also have high NAD^+^ activity, demonstrated a detectable level of NADP^+^-dependent GAPDH activity. In the holoenzyme wild-type GAPDH, residues G188 and P189 are located in the central part of the S-loop of each monomer near the active centre (Figure 5). They do not take part in the extensive hydrophobic cluster formation but instead participate in locally favor optimal binding of the NAD^+^ cofactor. It is known that proline and glycine are both helix breakers. Amino acid replacements in positions 188 and 189 possibly led to an increase in space in the coenzyme-binding pocket. This type of modification does not injure the active centre of the enzyme, maintains the high efficiency toward NAD^+^, and a high level of corresponding NAD^+^-dependent activity; at the same time, two mutant proteins also show NADP^+^-dependent activity, and for GapA^G188TP189K^, this is sufficient.

The combination of different variants of amino acid residues in positions 34, 188, and 189 did not provide the desired result—the creation of an enzyme with predominant NADP-dependent activity (Table 4 and Table 5). The mutant enzymes GapA^D34AG188N^, GapA^D34AG188VP189R^, and GapA^D34KG188TP189K^ showed a drastic drop in both activities; kinetic parameters of the protein GapA^D34AG188TP189K^ appeared to be very close to the mutant values with single substitution in the 34th position: GapA^D34A^ and GapA^D34K^. However, the mutant GapA^D34A G188TP189K^ is the most interesting among the mutant GAPDHs with a low level of dual NAD^+^/NADP^+^ activity due to the practically equal values of specific activity, Km, and specificity constant against both cofactors. The mutant GapA^G188TP189K^ is characterized with contrary futures: the highest among mutant enzyme levels for both activities, the best values of kcat/Km for both cofactors; however, all parameters for NAD^+^-dependent activity significantly exceed the corresponding indicators for NADP^+^-dependent activity. Thus, these mutant GAPDHs were chosen for further study.

### 3.5. l-Amino Acids Production by Strains with NAD^+^/NADP^+^-Dependent GAPDHs

As noted above, the NADP^+^-dependent GAPDH can increase the production of NADPH and, therefore, increase its entry into the amino acid biosynthetic pathways that use this cofactor (for example, l-aspartic and l-glutamic acid family pathways), thereby improving the accumulation of the corresponding amino acids by producer strains. This effect can manifest itself when cells grow on glucose; indeed, it was shown that the increased lysine yield by *C. glutamicum* cells was mainly due to the NADPH generated by the mutated GAPDH in EMP [12,22]. However, the effect may be more pronounced when cultivating on xylose because its assimilation occurs using the non-oxidative branch of PPP with the formation of two fewer molecules of NADPH than in the case of glucose. Therefore, we investigated the effect of changing the GAPDH cofactor specificity with the accumulation of l-lysine, l-threonine, and l-proline by the corresponding producing strains under conditions of growth on glucose and xylose as carbon sources.

The genes coding for wild-type GAPDH and mutant enzymes, GapA^D34AG188TP189K^ and GapA^G188TP189K^, were cloned into the middle-copy number vector, pMIV5JS, yielding plasmids pGAP-wt, pGAP-ATK, and pGAP-TK, respectively.

**Figure 6:**
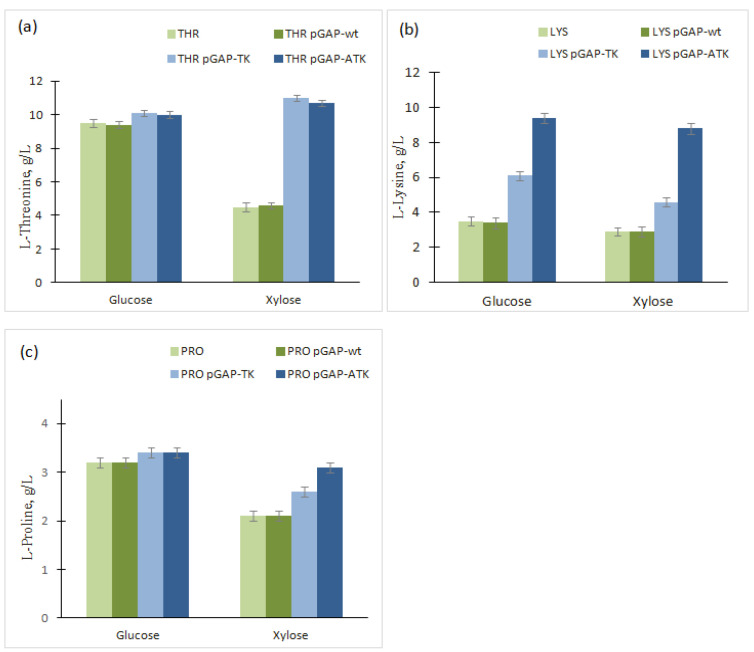
Extracellular accumulation of l-amino acids by *E. coli* strains overexpressing GAPDH of different cofactor specificities and grown on glucose or xylose as the carbon source. (**a**) l-threonine production (72 h of cultivation), (**b**) l-lysine production (48 h of cultivation), and (**c**) l-proline production (72 h of cultivation). The standard deviations from the means are indicated as error bars.

Model l-threonine-, l-lysine- and l-proline-producing strains (THR, LYS, and PRO, respectively) were transformed with each of these plasmids. The resulting strains were cultivated in media supplemented with glucose or xylose as the single carbon source. The sugar initially added to the medium was completely consumed within the time of cultivation in all samples. Accumulation of amino acids in culture broth increased for GAPDH dual specificity strains when grown on both sugars, although to a different extent (Figure 6).

The concentrations of l-lysine accumulated on glucose by the LYS/pGAP-TK and LYS/pGAP-ATK strains were 74% and 168% higher, respectively, than that of the control strain. Under the same conditions, the differences in the levels of l-threonine and l-proline production by pGAP-TK- and pGAP-ATK-harboring strains compared to the respective control strains were small (2–5%). However, the positive effect on the production of amino acid was much stronger when strains were cultivated on xylose. Strains LYS/pGAP-TK and LYS/pGAP-ATK produced 1.5- and 3.0-fold more l-lysine than LYS/pGAP-wt. The production of l-threonine by THR/pGAP-TK and THR/pGAP-ATK was 2.4-fold higher than that of the control strain harboring wild-type GAPDH. Finally, expression of mutant GAPDH led to an increase in the l-proline accumulation by the PRO-harboring pGAP-TK or pGAP-ATK plasmid, which increased compared to PRO/pGAP-wt by 24% and 48%, respectively.

We suggest that the more pronounced positive effect on the accumulation of l-lysine, l-threonine, and l-proline when cells are grown on xylose may be associated with higher NADPH deficiency for amino acid biosynthesis under these conditions compared to growth on glucose. The growth of all tested strains, including plasmid-free strains and those harboring plasmids with the wild-type and mutant *gap*A gene, was the same. Moreover, the cells of the producing strains overexpressing the wild-type *gap*A did not lead to an increase in the accumulation of the corresponding l-amino acid, indicating sufficient glycolitic NAD-dependent GAPDH activity. These data indirectly indicate that the increased l-amino acid accumulation by strains producing the mutant enzymes, GapA^D34AG188TP189K^ and GapA^G188TP189K^, is attributed to improvement in the intracellular NADPH pool due to activity of GAPDH with dual cofactor specificity and the new NADPH-generating property of the glycolytic pathway.

## 4. Discussion

It is known that the balance of the oxidation–reduction states of NAD^+^/NADH and NADP^+^/NADPH are crucial for both catabolism and anabolism. Some examples have illustrated the importance of redox balance and cofactor’s availability for producing fine chemicals, including amino acids, vitamins, and lipids [3,18,19]. In this study, replacement of native NAD^+^-dependent GAPDH activity with the NAD^+^/NADP^+^-dependent activity was performed based on 3D protein structure analysis, site-directed mutagenesis of amino acid residues 34, 188, and 189, and a combination of the most promising mutations. The construction of a set of *E. coli* GAPDHs with dual cofactor specificity that possesses different levels of both enzymatic activity and affinity against NAD^+^/NADP^+^ was demonstrated. As carbon of sugar inevitably passes through the GapA reaction regardless of whether it is utilized via glycolysis or via PPP, we hypothesized that the strains producing mutant GAPDH with dual cofactor specificity can generate more NADPH than the wild-type strains, as it was shown for *C. glutamicum* [22]. This assumption was indirectly confirmed by the results of our experiment with *E. coli* l-amino acid producers. Overproduction of mutant GAPDHs, GapA^D34AG188TP189K^ and GapA^G188TP189K^, in amino acid-producing *E. coli* cells led to considerably increased extracellular accumulation of l-lysine, l-threonine, and l-proline, whose biosynthesis requires a sufficient amount of NADPH. Thus, l-lysine accumulation increased 1.6- and 3.0-times on glucose and xylose, respectively. Note that, as in the work of Bommareddy et al., the mutant with a similar affinity for both coenzymes, GapA^D34A G188TP189K^, achieved the highest production of l-lysine. l-threonine and l-proline production on xylose grew by 2.4-fold and 48%, respectively. The more pronounced effect on xylose than on glucose can be explained by the higher deficiency of NADPH when grown on xylose. This means that the introduction of an enzyme with dual cofactor specificity, which allows sufficient NADPH supply irrespective of the flux distribution between glycolysis and the PPP, can overcome the sugar dependence problem. The increase in amino acid production also confirms that mutant enzymes can exhibit NADP^+^-dependent activity not only in vitro but also in vivo in cells during fermentation in the presence of both NAD^+^ and NADP^+^. The strategy of expressing NADP^+^-dependent GAPDH instead of the native NAD^+^-dependent enzyme can provide an additional source of NADPH during glycolysis, thus facilitating the biosynthesis of compounds through NADPH-dependent pathways. Similar strategies have resulted in increases in NADPH-dependent product synthesis in other organisms [7,8,12,22]. Mutant genes encoding enzymes with dual cofactor specificity expressed at the required level of expression can be used to construct producers of industrially significant substances. Our results not only reconfirm the importance of NADPH supply for efficient amino acid production but also demonstrate that this approach allows reconstruction of a functional glycolytic pathway with a new source of NADPH supply in *E. coli*.

## Figures and Tables

**Table 1:** *E. coli* strains used in this study.

Strain	Description	Source
MG1655	F^−^	CGSC 6300
YA1461	MG1655 *P_ltac_adhE*^E568K,I554S,E22G,M236V,A786V,Y461C^	[24]
YA1461ΔgapA::kan	P_ltac_adhE*; Δ*gapA::kan*	Current work
MG1655Cm-ymfR::gapA	*ymfR::gapA-cat*	Current work
MG1655Cm-ymfR::gapAΔgapA	*ymfR::gapA-cat*; Δ*gapA*	Current work
LYS (l-lysine producer)	WC196 Δ*cadA*; Δ*ldcC*; *P_ltac_xylAB*; pCABD2	FERM (BP-5252) (Patent USA 6,040,160)
PRO (l-proline producer)	702*ilvA*	VKPM ^a^ B-8012
THR (l-threonine producer)	MG1655 Δ*tdh*; *rhtA23*; pvic40	WO2009022754A

^a^ VKPM, The Russian National Collection of Industrial Microorganisms.

**Table 2:** Plasmids used in this study.

Plasmid	Antibiotic Resistance	Description	Source
pKD46	Amp	ori R101; *repA101*(ts); *P_araB_-gam-bet-exo*	[25]
pKK233-2	Amp	ori pBR322; *P_trc_lacZ′*; *rrnBT1T2*	Pharmacia, Sweden
pMW119-attL-Km-attR	Amp, Km	ori pSC101; *attLλ-kan-attRλ*	[26]
pMW119-attL-Cm-attR	Amp, Cm	ori pSC101; *attLλ-cat-attRλ*	[26]
pMIV5JS	Amp, Cm	ori pSC101; *attRµ*; *attLλ-cat-attRλ*; *attLμ*	EP1942183
pKK-gapA^wt^	Amp	ori pBR322; *P_trc_lacZ′*; *rrnBT1T2*; *gapA*^wt^	Current work
pKK-gapA^D34A^	Amp	ori pBR322; *P_trc_lacZ′*; *rrnBT1T2*; *gapA*^D34A^	Current work
pKK-gapA^D34P^	Amp	ori pBR322; *P_trc_lacZ′*; *rrnBT1T2*; *gapA*^D34P^	Current work
pKK-gapA^D34K^	Amp	ori pBR322; *P_trc_lacZ′*; *rrnBT1T2*; *gapA*^D34K^	Current work
pKK-gapA^D34L^	Amp	ori pBR322; *P_trc_lacZ′*; *rrnBT1T2*; *gapA*^D34L^	Current work
pKK-gapA^D34N^	Amp	ori pBR322; *P_trc_lacZ′*; *rrnBT1T2*; *gapA*^D34N^	Current work
pKK-gapA^G188N^	Amp	ori pBR322; *P_trc_lacZ′*; *rrnBT1T2*; *gapA*^G188N^	Current work
pKK-gapA^G188T P189K^	Amp	ori pBR322; *P_trc_lacZ′*; *rrnBT1T2*; *gapA*^G188T P189K^	Current work
pKK-gapA^G188V P189R^	Amp	ori pBR322; *P_trc_lacZ′*; *rrnBT1T2*; *gapA*^G188V P189R^	Current work
pKK-gapA^D34A G188N^	Amp	ori pBR322; *P_trc_lacZ′*; *rrnBT1T2*; *gapA*^D34A G188N^	Current work
pKK-gapA^D34A G188T P189K^	Amp	ori pBR322; *P_trc_lacZ′*; *rrnBT1T2*; *gapA*^D34AG188T P189K^	Current work
pKK-gapA^D34A G188V P189R^	Amp	ori pBR322; *P_trc_lacZ′*; *rrnBT1T2*; *gapA*^D34AG188V P189R^	Current work
pKK-gapA^D34K G188T P189K^	Amp	ori pBR322; *P_trc_lacZ′*; *rrnBT1T2*; *gapA*^D34KG188T P189K^	Current work
pKK-gapA^D34K G188V P189R^	Amp	ori pBR322; *P_trc_lacZ′*; *rrnBT1T2*; *gapA*^D34KG188V P189R^	Current work
pKK-gapA^D34A G188N^	Amp	ori pBR322; *P_trc_lacZ′, rrnBT1T2*; *gapA*^D34A G188N^	Current work
pKK-gapA^D34A G188T P189K^	Amp	ori pBR322; *P_trc_lacZ′*; *rrnBT1T2*; *gapA*^D34A G188T P189K^	Current work
pKK-gapA^D34A G188V P189R^	Amp	ori pBR322; *P_trc_lacZ′*; *rrnBT1T2*; *gapA*^D34A G188V P189R^	Current work
pvic40	Str	ori pRSF1010; thrA*BC	US 5,705,371
pCABD2	Str	ori pRSF1010; lysC*80; dapA*24; *dapB*; *DDH*	US 6,040,160

**Table 3:** NAD^+^-dependent GAPDH activity in *E. coli* strains.

Strain	GAPDH, μmol min^−1^ mg^−1^
MG1655	0.49 ± 0.03
YA1461 *	0.43 ± 0.2
MG1655 Cm-gapA::ymfR	0.88 ± 0.05
MG1655 Cm-gapA::ymfR ΔgapA::kan	0.40 ± 0.02
YA1461ΔgapA::kan **	<0.005

Cells were grown in M9 medium with 0.4% glucose and 5% LB for 5 h at 37 °C; * Cells were grown in M9 with 2% ethanol for 7 h at 37 °C; ** Cells were grown in M9 medium with 0.4% glucose, 2% ethanol, and 0.1% yeast extract for 7 h at 37 °C.

**Table 4:** NAD^+^/NADP^+^-dependent GAPDH activity in plasmid-harboring *E. coli* YA1461ΔgapA::kan.

Plasmid	GAPDH Specific Activity, μmol min ^−1^ mg ^−1^	
	NAD^+^	NADP^+^
no	0.83 ± 0.05	<0.005
pKK-gapA^wt^	19.88 ± 0.12	<0.01
Mutations in 34 position		
pKK-gapA^D34A^	1.95 ± 0.07	0.69 ± 0.015
pKK-gapA^D34P^	0.04 ± 0.002	0.034 ± 0.003
pKK-gapA^D34K^	0.23 ± 0.015	0.061 ± 0.004
pKK-gapA^D34L^	0.037 ± 0.004	0.022 ± 0.003
pKK-gapA^D34N^	0.043 ± 0.003	<0.01
Mutation in 188–189 positions		
pKK-gapA^G188N^	31.19 ± 0.2	<0.01
pKK-gapA^G188T P189K^	33.38 ± 0.24	5.27 ± 0.11
pKK-gapA^G188V P189R^	19.90 ± 0.11	0.21 ± 0.08
Mutations in 34, 188 and 189 positions		
pKK-gapA^D34A G188N^	0.50 ± 0.06	0.28 ± 0.04
pKK-gapA^D34A G188T P189K^	2.09 ± 0.11	2.18 ± 0.09
pKK-gapA^D34A G188V P189R^	0.099 ± 0.004	0.091 ± 0.006
pKK-gapA^D34K G188T P189K^	0.139 ± 0.006	0.104 ± 0.005
pKK-gapA^D34K G188V P189R^	21.60 ± 0.10	3.76 ± 0.06

**Table 5:** Kinetic characteristics of GAPDHs.

Protein	NAD^+^			NADP^+^		
	K_m_, mM	kcat, s^−1^	kcat/Km, mM^−1^s^−1^	K_m,_ mM	kcat, s^−1^	kcat/Km, mM^−1^s^−1^
GapA^wt^	0.028 ± 0.003	153.6 ± 3.4	5500	nd	nd	nd
GapA^D34A^	3.0 ± 0.4	36.6 ± 2.6	12.2	2.0 ± 0.3	22.8 ± 0.5	11.4
GapA^D34K^	5.1 ± 0.8	81.5 ± 7.8	16.0	1.1 ± 0.2	11.1 ± 0.8	10.0
GapA^D34L^	3.9 ± 0.3	25.6 ± 1.0	6.6	1.7 ± 0.2	4.6 ± 0.2	2.7
GapA^D34P^	4.3 ± 0.6	34.1 ± 2.6	7.9	1.7 ± 0.3	2.5 ± 0.2	1.5
GapA^G188T P189K^	0.056 ± 0.005	337.5 ± 5.1	6030	1.1 ± 0.2	85.7 ± 3.1	77.9
GapA^D34A G188T P189K^	1.9 ± 0.02	20.2 ± 2.3	10.6	1.8 ± 0.3	18.6 ± 2.1	10.1

## Data Availability

The analyzed data presented in this study are included within this article. Further data are available on reasonable request from the corresponding author.

## Supplementary Materials

The following supporting information can be downloaded at: https://www.mdpi.com/article/10.3390/microorganisms10050976/s1, Figure S1: SDS-PAGE analysis of the results of fractional precipitation. Strain YA1461/pKK-gapA^wt^, Figure S2: Alignment of the sequence of GAPDH relevant for cofactor specificity predictions, Figure S3: SDS-PAGE analysis of total proteins of *E. coli* plasmid-harboring strains YA1461ΔgapA::kan, Table S1: Primers used in this study, Table S2: Growth of strain YA1461ΔgapA::kan on media with different carbon sources.
