# Supplementary material for: Engineering of Escherichia coli Glyceraldehyde-3-Phosphate Dehydrogenase with Dual NAD+/NADP+ Cofactor Specificity for Improving Amino Acid Production
Source: Microorganisms. 2022 May 6;10(5):976. doi: 10.3390/microorganisms10050976 (PMC9145139; doi:10.3390/microorganisms10050976)
Supplement: Supplementary file 1 [file microorganisms-10-00976-s001.zip › microorganisms-1684931-supplementary.pdf]

Supplementary Materials.

Table S1. Primers used in the study.

Primers	Sequence
P1	5'atgactatcaaagtaggtatcaacggttttggccgtatcgctcaagttagtataaaaaagct3’
P2	5'agatgtgagcgatcaggtccagaactttgttggagttgaagcctgctttttatactaag3’
P3	5'attccgcttgacgctgcg3'
P4	5'tcttaatcatgacgcagtc3'
P5	5’ttgagtcctacgggatccgctcgctttacctgaggtaattcgctcaagttagtataaaaaagctgaac3’
P6	5'gtgttaatgttttgttagaatcagtcactgaagcctgctttttatactaagttggc3’
P7	5’gccaacttagtataaaaaagcaggcttcagtgactgattctaacaaaacattaacac3’
P8	5’caccggagctccttgcatcgtcagttgtcgtgtactgcaccaggcacagtgtcatctcaacttatttgg3’
P9	5’catcatgacggcaatcccatcatg3’
P10	5’gctcttcagtatcaaatccgcctg3’
P11	5’gttctgacatcgagatcgttgcaatcaacnnnctgttagacgctgattacatggcataca3’
P12	5’tgtatgccatgtaatcagcgtctaacagnnngttgattgcaacgatctcgatgtcagaac3’
P13	5’cataagcttttatttggagatgtgagcgatcaggt3’
P14	5’catggatcctgactgattctaacaaaacattaac3’
P15	5’gttgataccaaatctcacaaagactggcgcggc3’
P16	5’gtgagatttggtatcaacggttttctgagtagcgg3’
P17	5’gttgatgttcgttctcacaaagactggcgcggc-3’
P18	5’gtgagacggattatcaacggttttctgagtagcgg3’
P19	5’cagaaaaccgttgatggcccg3’
P20	5’cagaaaaccgttgataccaaa3’
P21	5’cagaaaaccgttgatgttcgt3’

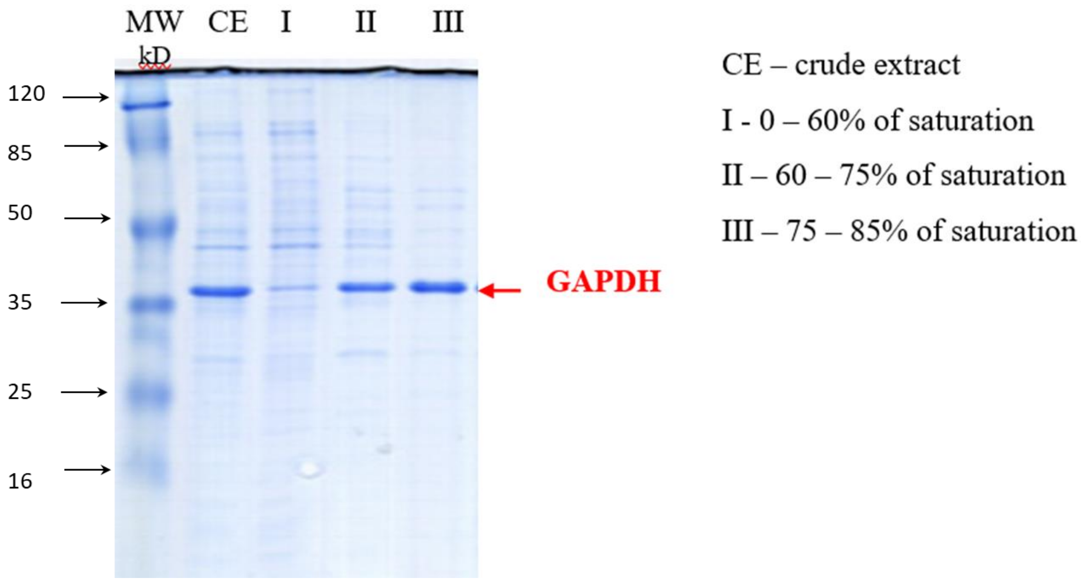

Figure S1. SDS-PAGE analysis of the results of fractional precipitation.

Strain YA1461- pKK-gapA^{wt}

Table S2. Growth of strain YA1461ΔgapA::kan on media with different carbon sources.

Media							OD_{600}
LB							0.2±0.05
M9							
Glycerol	Glucose	Xylose	Ethanol	Succinate	Glutamate	Yeast ext.	
+	-	-	-	-	-	-	0.1±0.05
+	-	-	+	+	+	+	3.5±0.20
+	-	-	+	+	+	-	1.8±0.10
+	-	-	-	+	+	+	1.1±0.05
-	-	-	+	+	+	+	4.1±0.25
+	-	-	+	-	+	+	3.9±0.20
+	-	-	+	+	-	+	3.4±0.10
-	+	-	-	-	-	-	0.1±0.05
-	+	-	+	+	+	+	4.8±0.15
-	+	-	-	+	+	+	1.2±0.10
-	+	-	+	-	-	+	5.6±0.3
-	-	+	+	+	+	+	4.6±0.20
-	-	+	-	+	+	+	1.2±0.10

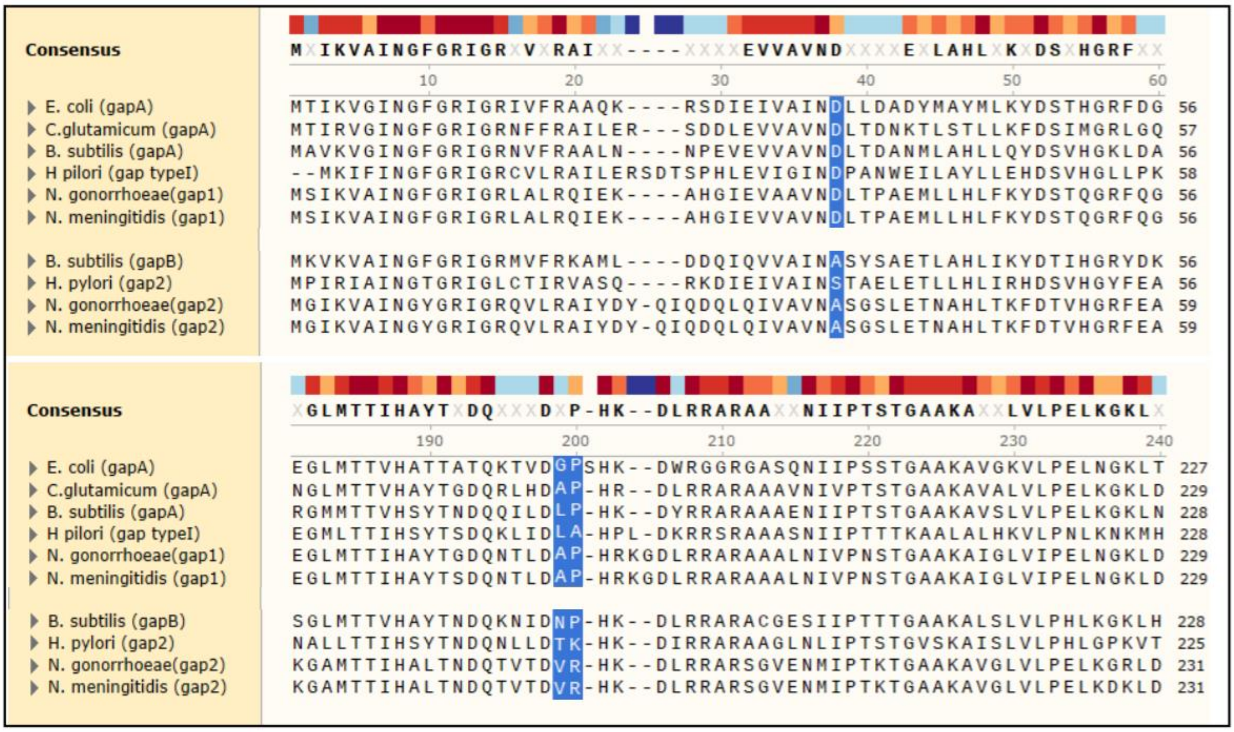

Figure S2. Alignment of the sequence of GAPDH relevant for cofactor specificity predictions.

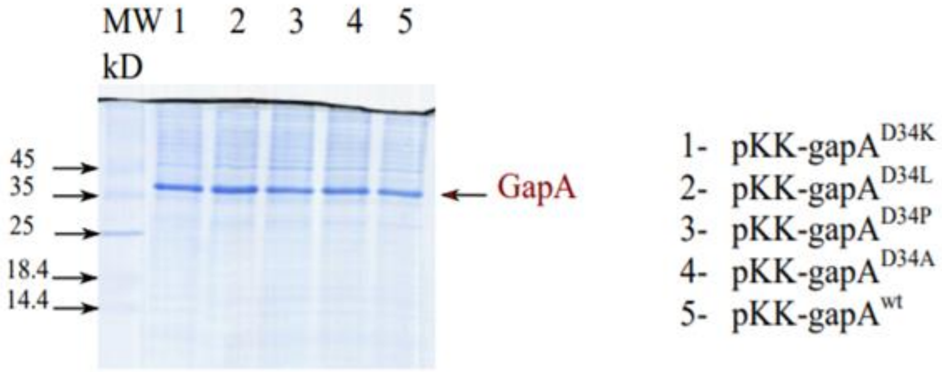

Figure S3. SDS-PAGE analysis of total proteins of *E. coli* plasmid-harboring strains YA1461ΔgapA::kan.
